# Carnosol suppresses patient-derived gastric tumor growth by targeting RSK2

**DOI:** 10.18632/oncotarget.24409

**Published:** 2018-02-06

**Authors:** Li Wang, Yujuan Zhang, Kangdong Liu, Hanyong Chen, Ran Yang, Xiaoli Ma, Hong-Gyum Kim, Ann M. Bode, Dong Joon Kim, Zigang Dong

**Affiliations:** ^1^ China–US (Henan) Hormel Cancer Institute, Henan, China; ^2^ The Pathophysiology Department, The School of Basic Medical Sciences, Zhengzhou University, Zhengzhou, Henan, China; ^3^ The Affiliated Cancer Hospital, Zhengzhou University, Zhengzhou, Henan, China; ^4^ The Collaborative Innovation Center of Henan Province for Cancer Chemoprevention, Zhengzhou, China; ^5^ The Hormel Institute, University of Minnesota, Austin, MN, USA

**Keywords:** carnosol, RSK2, gastric cancer, patient-derived tumor xenograft

## Abstract

Carnosol is a phenolic diterpene that is isolated from rosemary, sage, and oregano. It has been reported to possess anti-oxidant, anti-inflammatory, and anti-cancer properties. However, the molecular mechanism of carnosol's activity against gastric cancer has not been investigated. Herein, we report that carnosol is an RSK2 inhibitor that attenuates gastric cancer growth. Carnosol reduced anchorage-dependent and -independent gastric cancer growth by inhibiting the RSKs-CREB signaling pathway. The results of *in vitro* screening and cell-based assays indicated that carnosol represses RSK2 activity and its downstream signaling. Carnosol increased the G2/M phase and decreased S phase cell cycle and also induced apoptosis through the activation of caspases 9 and 7 and inhibition of Bcl-xL expression. Notably, oral administration of carnosol suppressed patient-derived gastric tumor growth in an *in vivo* mouse model. Our findings suggest that carnosol is an RSK2 inhibitor that could be useful for treating gastric cancer.

## INTRODUCTION

Gastric cancer is one of the most common malignant cancers. It is the second most frequently diagnosed cancer and the third leading cause of cancer-related mortality in the world [1]. Although the mortality rate has declined due to improved prevention and treatment, the five-year survival rate of gastric cancer patients is approximately 15 to 35% [2]. Early detection or diagnosis of gastric cancer is difficult and, thus, most patients are diagnosed at advanced stages. Other difficulties that gastric cancer patients experience include high recurrence rates, metastasis and development of resistance to chemotherapy [3, 4].

The Ras/MAPK (mitogen-activated protein kinase) pathway plays a central role in transducing extracellular signals to intracellular target proteins involved in cell growth, proliferation, cell motility and apoptosis [5]. The RSK (90 kDa ribosomal S6 kinase) is a serine/threonine kinase that is a downstream effector of the Ras/ERKs (extracellular signal regulated kinases) signaling pathway [6]. The RSK family contains four isoforms (RSK1 to 4) and is classified structurally as the RSK-like protein kinase/mitogen and stress activated kinase-1 (RLPK/SSK1) and RSK-B (MSK2) [7]. The structure of the RSKs contains two functional kinase domains. The N-terminal kinase domain (NTKD) of RSKs is responsible for downstream substrate phosphorylation, whereas the C-terminal kinase domain (CTKD) of RSKs activates the NTKD through autophosphorylation of the hydrophobic motif [8–10]. *RSK1* mRNA is predominantly found in the lung, kidney and pancreas, whereas both *RSK2* and *RSK3* mRNA are more abundant in skeletal muscle, heart and pancreas [7]. RSKs regulate diverse cellular processes including growth, survival and motility, and phosphorylate downstream targets such as CREB [11], c-Fos [12], Bad [13], GSK3β [7], ATF1 [14] and histone H3 [15]. Therefore, RSK is a multifunctional effector of the MAPK signaling pathway as well as an important therapeutic target in various cancers.

Many phytochemicals have been found to possess preventive or therapeutic activities against cancer, presumably without causing severe side effects compared to conventional treatments [16, 17]. A large amount of cancer research has focused on the identification and development of new chemotherapeutic agents that are derived from plants. A number of different phytochemicals found in the diet have been reported to exert inhibitory effects against various types of cancer cells *in vitro* and *in vivo* [18]. For example, rosemary contains various phenolic diterpenes, such as carnosic acid, carnosol and rosmarinic acid, which have been reported to exert antioxidant [19], anti-inflammatory [20], antidiabetic [21] and anticarcinogenic activities [22]. Carnosol is a natural polyphenol compound and is isolated from rosemary, sage and oregano [23, 24]. The anticancer properties of carnosol include inhibition of cell proliferation, induction of cell apoptosis, and reduction of cell motility mediated through various signaling proteins such as p38 MAP kinase, ERKs, p53, AMPK, activation of caspase 9, and 3, STAT3, NF-κB and COX2 [25–28]. Previous studies suggest that carnosol strongly inhibits TPA/DMBA-induced skin carcinogenesis by suppressing ornithine decarboxylase expression [29]. Additionally, the anti-cancer activity of carnosol in various animal models has also been reported in a prostate cancer xenograft model [30], in colon carcinogenesis using the APC^min^ mouse model [31], as well as in carbon tetrachloride-induced hepatocellular carcinogenesis [32].

Although previous studies suggest that carnosol might be useful in cancer prevention and therapy, the direct molecular targets of carnosol in gastric cancer have not yet been investigated. In this study, we identified direct targets of carnosol and investigated its efficacy against gastric cancer *in vitro* and *in vivo*. Herein, we report that carnosol is a potent RSK2 inhibitor that augments the efficacy of gastric cancer treatment.

## RESULTS

### Carnosol suppresses gastric cancer cell growth

**Figure 1:**
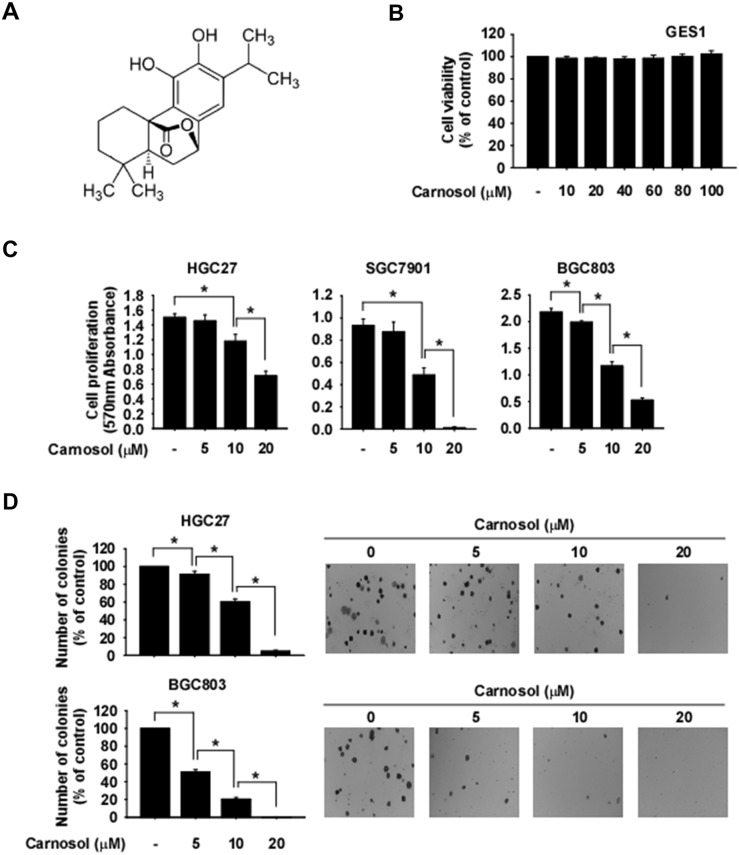
Anti-cancer effects of carnosol **(A)** Chemical structure of carnosol. **(B)** Effect of carnosol on the viability of normal gastric cells. Cells were treated with carnosol for 48 h. **(C)** Effect of carnosol on gastric cancer cell growth. Cells were treated with carnosol at various concentrations and then incubated for 72 h. For (B and C), cell viability and growth were measured at an absorbance of 570 nm. **(D)** The effect of carnosol on anchorage-independent growth of gastric cancer cells. Cells were treated with carnosol and incubated for 2 weeks. Colonies were counted using a microscope and the Image-Pro PLUS (v.6) computer software program. All data are shown as means ± S.D. of triplicate values from 3 independent experiments and the asterisk (^*^) indicates a significant (*p* < 0.05) inhibitory effect of carnosol.

Carnosol is an ortho-diphenolic diterpene compound with an abietane carbon skeleton (Figure 1A). To evaluate the effect of carnosol on cytotoxicity, we treated GES1 normal gastric epithelial mucosa cells with this compound. Results showed that carnosol had no cytotoxic effects on GES1 cells (Figure 1B). To determine whether phosphorylated RSK is differentially expressed in normal gastric or gastric cancer cells, we performed Western blot analysis. Results showed that gastric cancer cells highly express phosphorylated RSK compared with normal gastric cells (Supplementary Figure 1). We next determined whether carnosol could affect gastric cancer cell growth. HGC27, SGC7901 or BGC803 gastric cancer cells were treated with various concentrations of carnosol. Results indicated that carnosol significantly inhibited both anchorage-dependent (Figure 1C) and anchorage-independent (Figure 1D) gastric cancer cell growth in a dose-dependent manner.

### Carnosol is a potent RSK2 inhibitor

**Figure 2:**
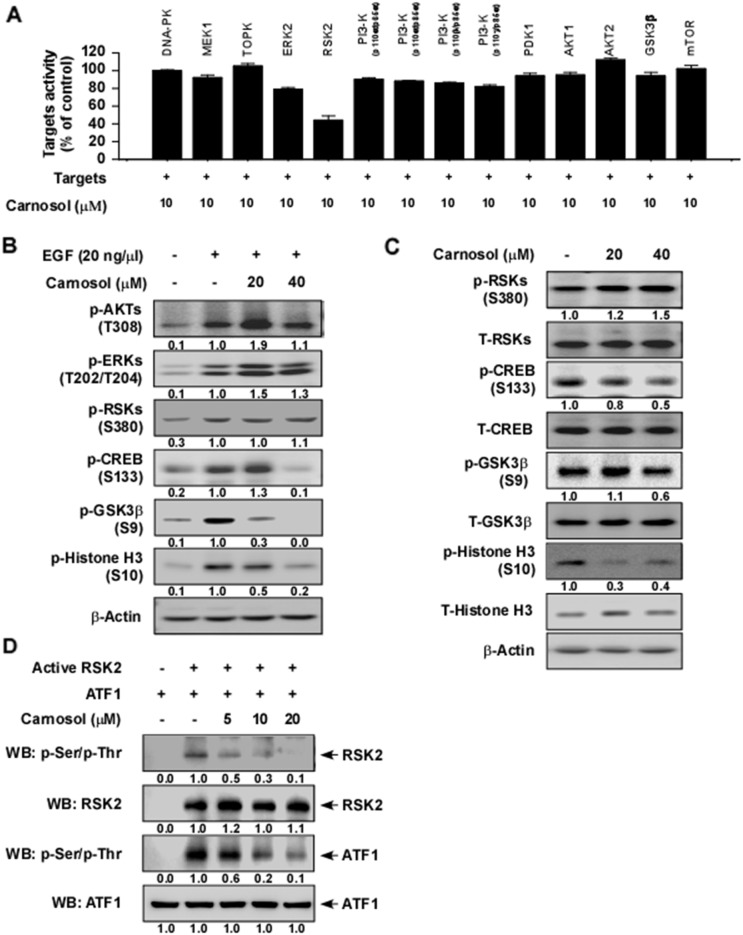
Carnosol is a potent RSK2 inhibitor **(A)** Screening ofthe effect of carnosol on various kinases. The effect of carnosol on kinase activity of DNA-PK, MEK1, TOPK, ERK2, RSK2, PI3K (p110α/p65α), PI3-K (p110α/p85α), PI3-K (p110β/p85α), PI3-K (p110γ/p85α), PDK1, AKT1, AKT2, GSK3β or mTOR was determined using active kinases and the specific substrate for each kinase. **(B)** Effect of carnosol on EGF-induced activation of various signaling proteins in JB6 cells. Serum-starved (0.1% FBS; 24 h) JB6 cells were treated with various doses of carnosol for 1.5 h followed by treatment with EGF for 30 min. **(C)** Effect of carnosol on various signaling proteins in SGC7901 gastric cancer cells. Cells were treated with carnosol at 20 or 40 μM for 3 h and then signaling pathway proteins were examined by Western blotting. **(D)** Carnosol suppresses RSK2 kinase activity in a dose-dependent manner. The effect of carnosol on RSK2 activity was assessed by an *in vitro* kinase assay using RSK2 (active, 300 ng) and ATF1 (substrate, 300 ng) proteins. The effect of carnosol on RSK2 activity was determined by Western blotting. For (B–D) data, similar results were obtained from 3 independent experiments.

To identify potential molecular targets of carnosol, we used *in vitro* kinase assays to screen the effect of carnosol against 14 different kinases. The results indicated that 10 μM carnosol strongly suppressed RSK2 activity, but had little effect on any other kinase (Figure 1A). We next determined whether carnosol could affect RSK2 downstream signaling. Following serum starvation for 48 h, JB6 cells were treated with carnosol for 1 h before treatment with epidermal growth factor (EGF) for 30 min. Results indicated that phosphorylation of CREB, GSK3β and histone H3 was strongly inhibited by carnosol treatment but phosphorylation of RSKs was not affected (Figure 2B). In addition, SGC7901 gastric cancer cells were treated with carnosol for 1 h and various signaling molecules were analyzed by Western blot. Results indicated that phosphorylation of CREB, GSK3β and histone H3 was substantially inhibited by carnosol treatment, whereas other signaling molecules were not affected (Figure 2C). To confirm whether carnosol has an effect on RSK2 signaling, we performed an *in vitro* kinase assay using a recombinant active RSK2 protein. These results showed that carnosol exerted strong dose-dependent inhibitory effects against RSK2 autophosphorylation and phosphorylation of its substrate, ATF1 (Figure 2D).

### Carnosol directly binds with RSK2

**Figure 3:**
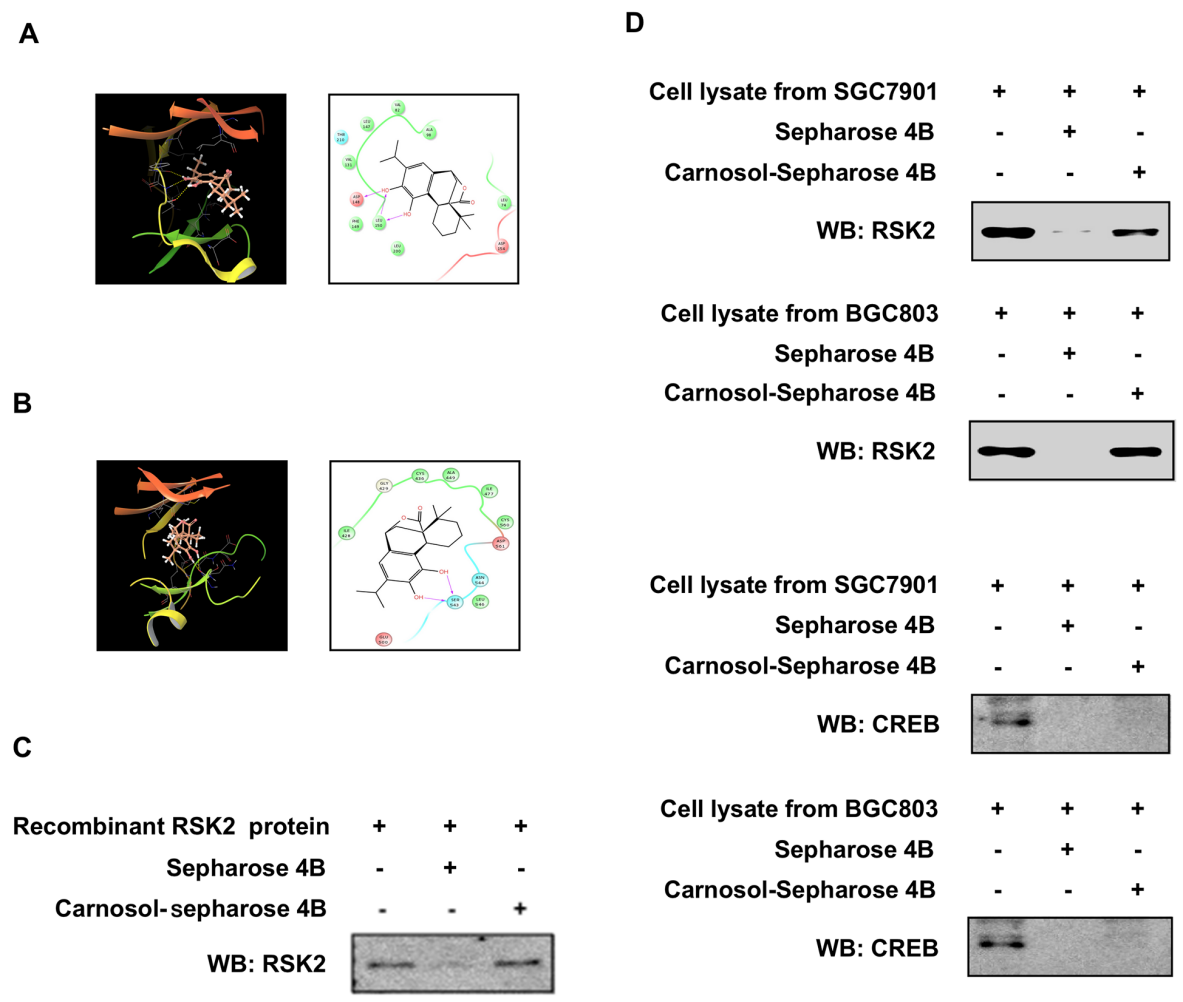
Carnosol directly binds to RSK2 **(A)** Modeling of carnosol binding with the N terminal domain (NTD) at the ATP binding pocket (i) of RSK2. Ligand Interaction Diagram (LID) of the binding (*right panel*). **(B)** Modeling of carnosol binding with the C terminal domain (CTD) at the ATP binding pocket (*left pane*l) of RSK2. Ligand Interaction Diagram (LID) of the binding (*right panel*). The NTD RSK2 and CTD RSK2 structures are shown inas ribbon representation and carnosol is shown as stick representation. Carnosol directly binds to RSK2 using a **(C)** recombinant protein or **(D)** gastric cancer cell lysates. The recombinant protein or cell lysate was incubated with carnosol-conjugated Sepharose 4B beads or with Sepharose 4B beads alone. The pulled down proteins were analyzed by Western blotting. Similar results were obtained from 3 independent experiments.

To further study the potential interaction between carnosol and RSK2, we created a computational docking model (using several protocols in the Schrödinger Suite 2016) of carnosol binding at the ATP pocket of NTD RSK2 and CTD RSK2, respectively. The computational docking model results indicated that carnosol formed several contacts with NTD RSK2 and CTD RSK2 at their respective ATP binding pockets (Figure 3A, 3B). Images were generated with the UCSF Chimera software program [34]. Next, to confirm the computational docking results, we performed *in vitro* pull-down assays with carnosol-conjugated Sepharose 4B beads (or Sepharose 4B beads as a negative control) and a recombinant RSK2 protein or SGC7901 and BGC803 gastric cancer cell lysates. Results indicated that carnosol directly binds to RSK2, but not with CREB in cells (Figure 3C, 3D).

### Carnosol induces G2/M phase cell cycle arrest

**Figure 4:**
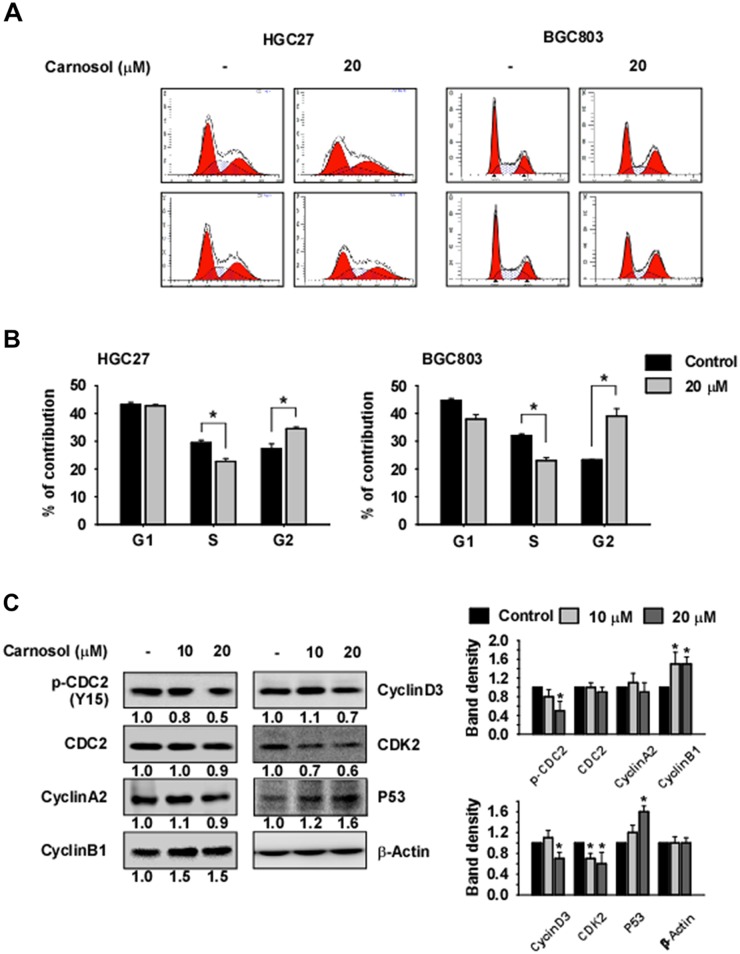
Carnosol induces G2/M phase arrest **(A-B)** The effect of carnosol on cell cycle was examined in HGC27 or BGC803 gastric cancer cells. Cells were synchronized by serum starvation for 24 h and treated with serum and/or carnosol for 24 h in 10% serum and medium. Cells were stained with propidium iodide(PI) and cell cycle was analyzed by Fluorescence Activated Cell Sorting (FACS). **(C)** The effect of carnosol on cell cycle marker proteins was determined. Synchronized cells were treated with serum and/or carnosol for 24 h in 10% serum and analyzed by Western blotting. Band density was measured using the Image J (NIH) software program. For (A–C), similar results were observed from 3 independent experiments.

To evaluate the effect of carnosol on cell cycle in HGC27 or BGC803 gastric cancer cells, we performed flow cytometry (FACS) analysis. Cells were synchronized by serum starvation for 24 h and cell cycle was released with serum with or without carnosol for 24 h. The results indicated that carnosol induces accumulation of cells in the G2/M phase and reduces the number of cells in S phase (Figure 4A, 4B). We also examined the effect of carnosol on cell cycle marker protein expression. Results indicated that expression of cyclin B1 and p53 was increased by carnosol treatment and expression of phosphorylated CDC2 and CDK2 was decreased (Figure 4C).

### Carnosol augments gastric cancer cell apoptosis

**Figure 5:**
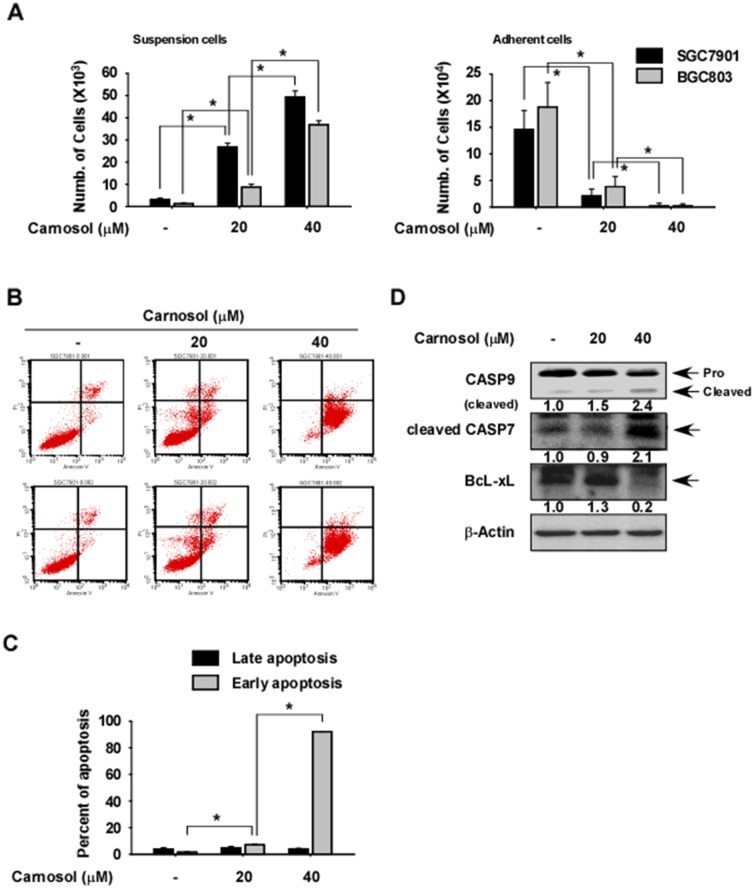
Effect of carnosol on gastric cancer cell apoptosis **(A)** Carnosol induces cell death. Cells were seeded in a 6-well plate and treated with carnosol at the indicated doses for 72 h. The number of suspended or attached cells was determined using a hematocytometer. **(B–C)** Carnosol induces apoptosis. Cells were seeded with carnosol in medium supplemented with10% FBS and then incubated for 72 h. Cells were stained with annexin V and propidium iodide(PI) and apoptosis was determined by Fluorescence Activated Cell Sorting (FACS). For (A–C), data are shown as mean values ± S.D. (n = 3) and the asterisk (^*^) indicates a significant (*p* < 0.05) difference compared to untreated control cells. **(D)** Carnosol strongly induces expression of apoptotic marker proteins. Cells were treated with carnosol for 72 h and the levels of caspase 9, cleaved caspase 7 and BcL-xL proteins were determined by Western blotting using β-actin as the loading control. Similar results were observed from 3 independent experiments. Band density was measured using the Image J (NIH) software program.

To investigate the effect of carnosol on gastric cancer cell apoptosis, we measured the viability of carnosol by counting SGC7901 or BGC803 gastric cancer cells found in suspended (dead) and attached (live) fractions after treatment for 48 h with different doses of carnosol. The results indicated that the number of suspended cells was significantly increased in carnosol-treated cells compared with control (Figure 5A, *left panel*), while that of attached cells was significantly decreased in carnosol-treated cells (Figure 5A, *right panel*). To determine whether the increased cell death was due to apoptosis, we measured annexin V expression at 48 h after carnosol treatment and found a significantly higher level of early apoptosis compared to untreated control cells (Figure 5B-5C). Increased apoptosis was also confirmed by measuring the expression levels of pro-apoptotic or anti-apoptotic marker proteins and results indicated that cleaved caspase 9 and 7 were strongly induced by carnosol treatment and anti-apoptotic BcL-xL was markedly reduced (Figure 5D).

### Carnosol inhibits patient-derived gastric tumor growth *in vivo*

**Figure 6:**
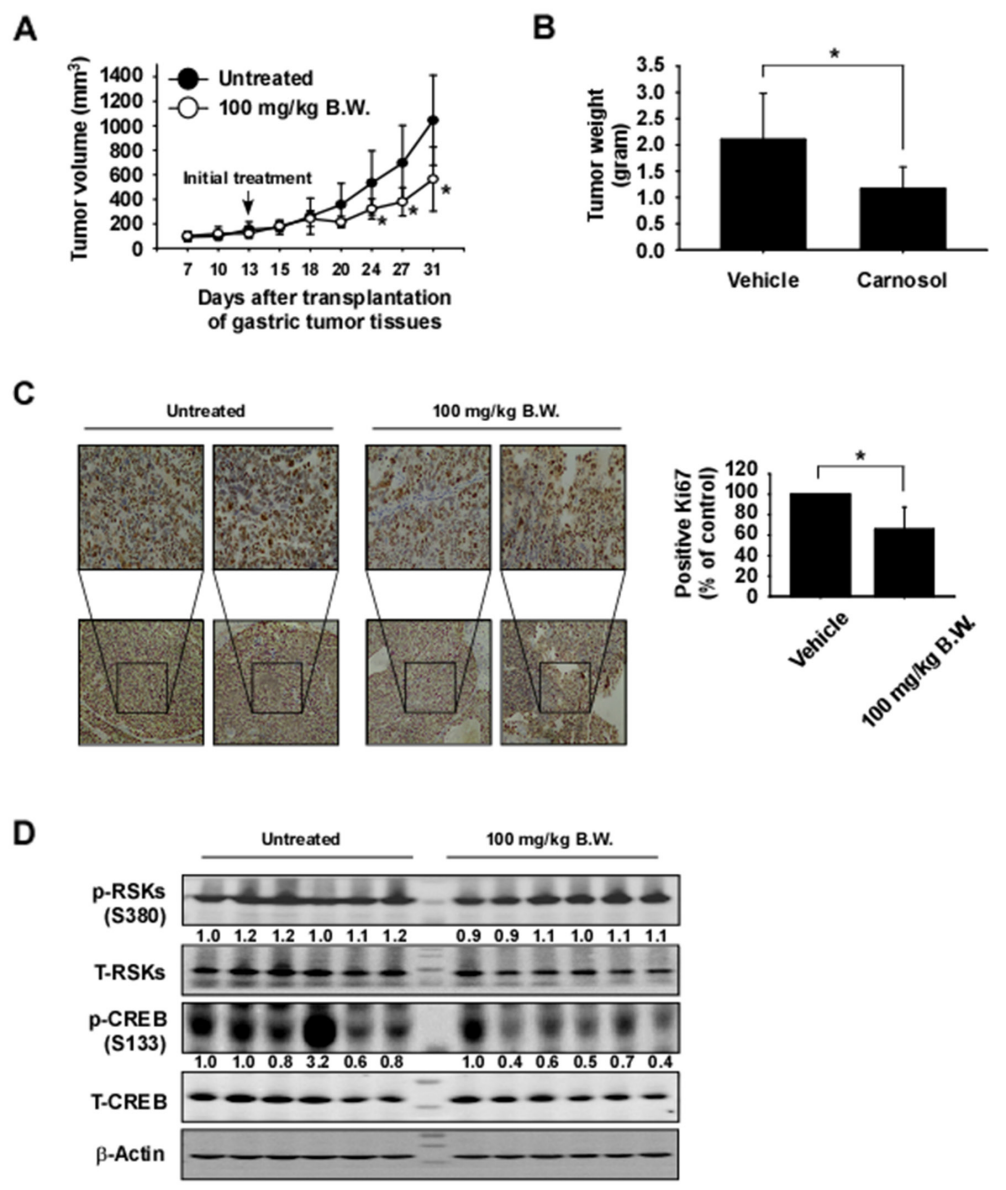
Carnosol attenuates gastric cancer patient-derived xenograft tumor growth *in vivo* Mice were divided into 2 groups to assess the effect of carnosol on gastric cancer patient-derived xenograft. Groups are as follows: 1) vehicle group or 2) administration of 100 mg/kg of carnosol. Mice were orally administered carnosol or vehicle 5 times a week for 18 days. **(A–B)** Carnosol inhibits gastric tumor growth. For (A–B), data are shown as means ± S.E. of values obtained from experiments. The asterisk (^*^) indicates a significant difference between tumors from untreated or treated mice as determined by *t* test (*p* < 0.05). **(C)** Immunohistochemistry analysis of tumor tissues. Tumor tissues from treated or untreated groups of mice were stained with anti-Ki-67. The number of Ki-67-positive stained cells (*right panel*) was quantified from immunohistochemistry results (n = 10; ^*^; *p* < 0.05). **(D)** Carnosol inhibits RSK-mediated CREB signaling protein expression in gastric tumor tissues. Tumor tissues from each group were immunoblotted with antibodies to detect RSKs, CREB, phosphorylated RSKs, phosphorylated CREB and β-actin. β-Actin was used to verify equal protein loading. Band density was measured using the Image J (NIH) software program.

To examine the anti-tumor activity of carnosol *in vivo*, patient-derived gastric tumor tissues were injected into the back of the neck of athymic nude mice. Mice were orally administrated carnosol at 100 mg/kg or vehicle 5 times a week over a period of 31 days. Results indicated that carnosol significantly decreased the volume and weight of gastric tumors relative to the vehicle-treated group (Figure 6A, 6B; *p* < 0.05). Additionally, mice tolerated treatment with carnosol without significant loss of body weight similar to the vehicle-treated group (Supplementary Figure 2A). We then examined the effects of carnosol on the Ki-67 tumor proliferation marker by using immunohistochemistry. The expression of Ki-67 was significantly decreased by treatment with carnosol (Figure 6C). Furthermore, to evaluate potential toxic effects of carnosol on tissue morphology, the liver and spleen tissues were stained with hematoxylin and eosin (H&E). Results indicated no obvious morphological changes (Supplementary Figure 2B, 2C). To validate the results of the *in vivo* PDX model, we investigated the effect of carnosol on RSK2-CREB signaling by Western blot analysis of PDX gastric tumor samples. The phosphorylation of CREB, a direct downstream protein of RSK2, was strongly inhibited in the carnosol-treated group but the expression of total CREB was relatively unchanged (Figure 6D).

## DISCUSSION

Gastric cancer remains one of the most common malignant diseases for which targeted therapies are emerging as treatment options. Promising target therapies by small molecule-induced blockade of the activity of specific oncogenic signaling pathways have been studied. Previous findings indicate that RAS/MAPK signaling is frequently activated by multiple types of genomic amplifications or mutations in gastric cancer [35]. Therefore, targeting of the RAS/MAPK signaling pathways could assist in the application of a molecular targeted therapy against gastric cancer. RSKs are downstream effectors of the MAPK signaling pathway and are heavily involved in tumorigenesis, survival and metastasis of various tumors mediated through their regulation of various substrates, including kinases and transcription factors [7, 8]. Additionally, RSKs are highly activated in various gastric cancer cell types (Supplementary Figure 1). Therefore, RSKs inhibitors might be promising therapeutic agents against gastric cancer. In the present study, we reported that carnosol strongly suppresses RSK2 kinase activity (IC_50_ ~ 5.5 μM; Figure 2D) and carnosol appears to be most potent against RSK2 compared with other kinases (Figure 2A). Additionally, carnosol significantly suppressed anchorage-dependent and -independent gastric cancer cell growth, but had little effect on normal gastric cell growth (Figure 1B-1D).

In previous studies, MAPK signaling was shown to play a role in the G1/S phase transition through cyclin D1 expression [36, 37] and also in the G2/M phase transition through the regulation of Myt1 [38] and the CDC2-cyclin B1 complex [39]. During the G2/M phase, the CDC2-cyclin B complex is in an inactive form because of the phosphorylation of CDC2 at Tyr15 by Wee1 and Myt1 [40, 41]. RSK directly phosphorylates the C-terminal of Myt1 and down-regulates its inhibitory activity against the CDC2-cyclin B complex [38, 41]. Therefore, we suggested that the inhibitory effect against RSK2 activity by carnosol treatment might reduce the phosphorylation of CDC2 at Tyr15 through regulation of Myt1 activity (Figure 4C). Additionally, RSK leads the G2/M phase transition by activating the phosphorylation of CDC25A and CDC25B [42]. Therefore, we determined whether G2/M phase is arrested by carnosol treatment in gastric cancer cells. Flow cytometric analysis revealed that carnosol induced G2/M phase arrest mediated through changes in cyclin B1, CDK2 and p53 expression as well as CDC2 activity (Figure 4A-4C).

RSK enhances cell survival through regulation of pro- or anti-apoptotic proteins including *bcl-2*, *bcL-xL, bad* and caspases activity [43, 44]. Therefore, we investigated whether carnosol could induce cellular apoptosis. Our results suggested that gastric cancer cells underwent increased apoptosis induced by carnosol treatment through the inhibition of the RSK/CREB signaling pathway (Figure 5A-5C). Blocking this signaling pathway resulted in increased cleavage of caspase-9 and -7 and decreased BcL-xL expression (Figure 5D).

Although advances have occurred in our understanding of human malignancies and molecular mechanisms of cancer biology, only 5% of anticancer drugs developed have been approved by the Food and Drug Administration (FDA). This is because the pre-clinical testing conducted did not consider tumor heterogeneity and human stromal microenvironmental conditions [45]. Therefore, studying tumor heterogeneity for improving drug efficacy is imperative. To overcome this limitation, researchers developed the patient-derived xenograft (PDX) model, which involves the direct implantation of a patient's primary tumor into compromised immune deficient mice [46]. In the current study, we investigated the antitumor effects of carnosol in gastric PDX models. Results indicated that oral administration of carnosol significantly inhibited gastric tumor growth by inhibiting RSKs/CREB signaling and was not toxic (Figure 6A, 6B and Supplementary Figure 2A-2C). In conclusion, our findings demonstrate that carnosol is a potent RSK2 inhibitor that could be useful for preventing or treating gastric cancers.

## MATERIALS AND METHODS

### Cell lines

BGC803 and SGC7901 human gastric cancer cells were purchased from KeyGEN BioTECH Corporation (Jiangsu, China). HGC27 human gastric cancer cells were purchased from the Chinese Academy of Sciences Typical Culture Collection (Shanghai, China). GESI human gastric cells were obtained from CHI Scientific, Inc. (Maynard, MA, USA). Enough frozen vials were available for each cell line to ensure that all cell-based experiments were conducted on cells that had been authenticated and in culture for a maximum of 8 weeks. JB6 mouse epidermal cells were cultured in minimal Eagle's medium (MEM) supplemented with 5% fetal bovine serum (FBS; Biological Industries, Cromwell, CT, USA) and 1% antibiotic-antimycotic. GES1 or BGC803 human gastric epithelial mucosa cells were cultured in Roswell Park Memorial Institute medium 1640 (RPMI1640) supplemented with 10% FBS and 1% antibiotic-antimycotic. HGC27 human gastric cancercells were cultured in Minimum Essential Medium with Earle's Balanced Salts (MEM/EBSS) supplemented with 1% non-essential amino acid (NEAA), 10% FBS and 1% antibiotic-antimycotic. SGC7901 human gastric cancercells were cultured in Dulbecco's Modified Eagle's Medium (DMEM) supplemented with 10% FBS and 1% antibiotic-antimycotic.

### Reagents and antibodies

Carnosol (purity: > 98% by HPLC) was purchased from Sigma-Aldrich (St. Louis, MO, USA). CNBr-Sepharose 4B beads were from GE Healthcare (Piscataway, NJ, USA). Antibodies to detect total ERKs, phosphorylated ERKs (T202/Y204), total RSK, phosphorylated RSK (S380), total AKT, phosphorylated AKT (S473), phosphorylated CREB (S133), total CREB, phosphorylated CDC2 (Y15), total CDC2, cyclin A2, cyclin B1, cyclin D1, p27(kip1), phosphorylated CDK2 (T160), total CDK2, caspase 9, cleaved caspase 7 and BcL-xL were obtained from Cell Signaling Technology (Beverly, MA, USA). The antibody to detect β-actin and p21 was from Santa Cruz Biotechnology (Santa Cruz, CA, USA). Active RSK2, and ATF1 (RSK2 substrate) human recombinant protein for kinase assays were purchased from SignalChem (Richmond, BC, Canada).

### Anchorage-independent cell growth

Cells (8 × 10^3^ per well) suspended in complete growth medium (Basal Medium Eagle; BME) supplemented with 10% FBS were added to 0.3% agar with or without different concentrations of carnosol in a top layer over a base layer of 0.6% agar with or without different concentrations of carnosol. The cultures were maintained at 37°C in a 5% CO_2_ incubator for 2 weeks and then colonies were counted under a microscope using the Image-Pro Plus software (v.6) program (Media Cybernetics, Rockville, MD, USA).

### Cell proliferation assay

To estimate viability, GES1 cells were seeded (3.5 × 10^3^ cells/well) in 96-well plates at 37°C in a 5% CO_2_ incubator and incubated for 24 h. HGC27, SGC7901 or BGC803cells were seeded (0.8 to 1 × 10^3^ cells per well) in 96-well plates and incubated for 24 h. Cells were treated with different concentrations of carnosol. After incubation for 48 h, 20 μl of MTT solution (Solarbio, Beijing, China) were added to each well and the cells were then incubated for 2 h at 37°C in a 5% CO_2_ incubator. The cell culture medium was removed and 200 μl of DMSO were added to each well and crystal formation was dissolved. Absorbance was measured at 570 nm.

### Pull-down assay using CNBr-carnosol-conjugated beads

A recombinant human RSK2 protein (200 ng) or total cell lysates (500 μg) were incubated with carnosol-sepharose 4B (or sepharose 4B only as a control) beads (50 μl, 50% slurry) in reaction buffer (50 mM Tris pH 7.5, 5 mM EDTA, 150 mM NaCl, 1 mM DTT, 0.01% NP40, 2 μg/mL bovine serum albumin). After incubation with gentle rocking overnight at 4°C, the beads were washed 5 times with buffer (50 mM Tris pH 7.5, 5 mM EDTA, 150 mM NaCl, 1 mM DTT, 0.01% NP40) and binding was visualized by Western blotting.

### Computational modeling of RSK2 with carnosol

To date, no full-length RSK2 crystal structure has been reported. For this study, the crystal structures of CTD (PDB ID:2QR8) and NTD of RSK2 (PDB ID:3G51) [9, 10] were retrieved from the Protein Data Bank (PDB) [33] and used in the computational study. The structures were prepared under the standard procedures of the Protein Preparation Wizard (Schrödinger Suite 2016). Hydrogen atoms were added consistent with a pH of 7 and all water molecules were removed. The ATP-binding site-based receptor grids of the RSK2 NTD and CTD were generated for docking. Carnosol was prepared using the LigPrep program of Schrödinger Suite 2016 for docking by default parameters. Then the docking of carnosol with NTD RSK2 and CTD RSK2 was accomplished with default parameters under the extra precision (XP) mode using the program Glide. Herein, we could get the best-docked representative structures.

### Cell cycle analysis

Cells were plated into 60-mm culture dishes (4 – 5 × 10^4^ cells/dish) and incubated for 24 h. Cells were synchronized by serum starvation for 24 h and treated with serum and/or carnosol for 24 h in 10% serum and medium. Cells were collected by trypsinization and washed with phosphate buffered saline (PBS) and then fixed in 1000 μl of 70% cold ethanol. After rehydration, cells were digested with RNase (100 μg/ml) and stained with propidium iodide (20 μg/ml). Propidium iodide staining was accomplished following the product instructions (Clontech, Palo Alto, CA, USA). The cells were analyzed by flow cytometry.

### Apoptosis assay

Cells were plated into 6 well culture dishes (8 × 10^4^ cells/well) and incubated for 24 h. Cells were treated with carnosol for 48 h in 10% serum-containing medium. Cells were collected by trypsinization and washed with phosphate buffered saline (PBS). Cells were stained with Annexin V (BioLegend, San Diego, CA, USA) and propidium iodide and then apoptosis was analyzed by flow cytomertry.

### Patient-derived xenograft (PDX) model

Female mice with severe combined immunodeficiency (SCID) [6–9 wk old] were maintained under “specific pathogen-free” conditions based on the guidelines established by Zhengzhou University Institutional Animal Care and Use Committee (Zhengzhou, Henan, China). A human tumor specimen of gastric tumor tissue was obtained from the Affiliated Cancer Hospital in Zhengzhou University, cut into pieces and implanted into the back of the neck of each mouse. Mice were divided into 2 groups of 10 animals each as follows: 1) untreated vehicle group and 2) 100 mg carnosol/kg of body weight. Carnosol or vehicle (5% DMSO in 10% tween 80) was orally administered 5 times per week. Tumor volume was calculated from measurements of 2 diameters of the individual tumor base using the following formula: tumor volume (mm^3^) = (length × width × height × 0.52). Mice were monitored until tumors reached 1.5 cm^3^ total volume, at which time mice were euthanized and tumors extracted.

### Hematoxylin and eosin staining and immunohistochemistry

Tumor, liver or spleen tissues from mice were embedded in paraffin blocks and subjected to hematoxylin and eosin (H&E) staining and immunohistochemistry (IHC). Tissue sections were deparaffinized and hydrated then permeabilized with 0.5% Triton X-100/1 × PBS for 10 min. After developing with 3, 3′-diaminobenzidine, the sections were counterstained with H&E. For IHC, sections were hybridized with the primary antibody (1:500) and a horse-radish peroxidase (HRP)-conjugated goat anti-rabbit or mouse IgG antibody was used as the secondary antibody. All sections were observed by microscope and the Image-Pro Plus software (v. 6) program (Media Cybernetics).

### Statistical analysis

All quantitative results are expressed as mean values ± S.D. or ± S.E. Significant differences were compared using the Student's *t* test or one-way analysis of variance (ANOVA). A *p* value of < 0.05 was considered to be statistically significant. The statistical package for social science (SPSS) for Windows (IBM, Inc.) was used to calculate the *p*-value to determine statistical significance.

## SUPPLEMENTARY MATERIALS FIGURES



## Abbreviations

RSK2: ribosomal protein S6 kinase 2
CREB: cAMP responsive element binding protein
PDX: patient-derived xenograft
AKT: V-akt murine thymoma viral oncogene homolog
ERKs: extracellular signal-regulated kinases
CDK2: cyclin dependent kinase 2
CDC2: cell division cycle protein 2.

## References

[1] Carcas LP (2014). Gastric cancer review. J Carcinog.

[2] Patru CL, Surlin V, Georgescu I, Patru E (2013). Current issues in gastric cancer epidemiology. Rev Med Chir Soc Med Nat Iasi.

[3] Cervantes A, Roda D, Tarazona N, Rosello S, Perez-Fidalgo JA (2013). Current questions for the treatment of advanced gastric cancer. Cancer Treat Rev.

[4] Oba K, Paoletti X, Bang YJ, Bleiberg H, Burzykowski T, Fuse N, Michiels S, Morita S, Ohashi Y, Pignon JP, Rougier P, Sakamoto J, Sargent D (2013). Role of chemotherapy for advanced/recurrent gastric cancer: an individual-patient-data meta-analysis. Eur J Cancer.

[5] Cargnello M, Roux PP (2011). Activation and function of the MAPKs and their substrates, the MAPK-activated protein kinases. Microbiol Mol Biol Rev.

[6] Pearson G, Robinson F, Beers Gibson T, Xu BE, Karandikar M, Berman K, Cobb MH (2001). Mitogen-activated protein (MAP) kinase pathways: Regulation and physiological functions. Endocr Rev.

[7] Lara R, Seckl MJ, Pardo OE (2013). The p90 RSK family members: Common functions and isoform specificity. Cancer Res.

[8] Anjum R, Blenis J (2008). The RSK family of kinases: Emerging roles in cellular signalling. Nat Rev Mol Cell Biol.

[9] Malakhova M, Tereshko V, Lee SY, Yao K, Cho YY, Bode A, Dong Z (2008). Structural basis for activation of the autoinhibitory C-terminal kinase domain of p90 RSK2. Nat Struct Mol Biol.

[10] Malakhova M, Kurinov I, Liu K, Zheng D, D’Angelo I, Shim JH, Steinman V, Bode AM, Dong Z (2009). Structural diversity of the active N-terminal kinase domain of p90 ribosomal S6 kinase 2. PLoS One.

[11] De Cesare D, Jacquot S, Hanauer A, Sassone-Corsi P (1998). Rsk-2 activity is necessary for epidermal growth factor-induced phosphorylation of CREB protein and transcription of c-fos gene. Proc Natl Acad Sci USA.

[12] Chen RH, Abate C, Blenis J (1993). Phosphorylation of the c-Fos transrepression domain by mitogen-activated protein kinase and 90-kDa ribosomal S6 kinase. Proc Natl Acad Sci USA.

[13] Tan Y, Ruan H, Demeter MR, Comb MJ (1999). p90(RSK) blocks bad-mediated cell death via a protein kinase C-dependent pathway. J Biol Chem.

[14] Liu K, Cho YY, Yao K, Nadas J, Kim DJ, Cho EJ, Lee MH, Pugliese A, Zhang J, Bode AM, Dong Z (2011). Eriodictyol inhibits RSK2-ATF1 signaling and suppresses EGF-induced neoplastic cell transformation. J Biol Chem.

[15] Strelkov IS, Davie JR (2002). Ser-10 phosphorylation of histone H3 and immediate early gene expression in oncogene-transformed mouse fibroblasts. Cancer Res.

[16] Hosseini A, Ghorbani A (2015). Cancer therapy with phytochemicals: Evidence from clinical studies. Avicenna J Phytomed.

[17] Russo M, Spagnuolo C, Tedesco I, Russo GL (2010). Phytochemicals in cancer prevention and therapy: truth or dare?. Toxins (Basel).

[18] Lee KW, Bode AM, Dong Z (2011). Molecular targets of phytochemicals for cancer prevention. Nat Rev Cancer.

[19] Raskovic A, Milanovic I, Pavlovic N, Cebovic T, Vukmirovic S, Mikov M (2014). Antioxidant activity of rosemary (Rosmarinus officinalis L.) essential oil and its hepatoprotective potential. BMC Complement Altern Med.

[20] Takaki I, Bersani-Amado LE, Vendruscolo A, Sartoretto SM, Diniz SP, Bersani-Amado CA, Cuman RK (2008). Anti-inflammatory and antinociceptive effects of rosmarinus officinalis L. essential oil in experimental animal models. J Med Food.

[21] Bakirel T, Bakirel U, Keles OU, Ulgen SG, Yardibi H (2008). *In vivo* assessment of antidiabetic and antioxidant activities of rosemary (Rosmarinus officinalis) in alloxan-diabetic rabbits. J Ethnopharmacol.

[22] Ngo SN, Williams DB, Head RJ (2011). Rosemary and cancer prevention: Preclinical perspectives. Crit Rev Food Sci Nutr.

[23] Aruoma OI, Halliwell B, Aeschbach R, Loligers J (1992). Antioxidant and pro-oxidant properties of active rosemary constituents: Carnosol and carnosic acid. Xenobiotica.

[24] Horiuchi K, Shiota S, Kuroda T, Hatano T, Yoshida T, Tsuchiya T (2007). Potentiation of antimicrobial activity of aminoglycosides by carnosol from Salvia officinalis. Biol Pharm Bull.

[25] Lo AH, Liang YC, Lin-Shiau SY, Ho CT, Lin JK (2002). Carnosol, an antioxidant in rosemary, suppresses inducible nitric oxide synthase through down-regulating nuclear factor-kappaB in mouse macrophages. Carcinogenesis.

[26] Subbaramaiah K, Cole PA, Dannenberg AJ (2002). Retinoids and carnosol suppress cyclooxygenase-2 transcription by CREB-binding protein/p300-dependent and -independent mechanisms. Cancer Res.

[27] Johnson JJ, Syed DN, Heren CR, Suh Y, Adhami VM, Mukhtar H (2008). Carnosol, a dietary diterpene, displays growth inhibitory effects in human prostate cancer PC3 cells leading to G2-phase cell cycle arrest and targets the 5′-AMP-activated protein kinase (AMPK) pathway. Pharm Res.

[28] Park KW, Kundu J, Chae IG, Kim DH, Yu MH, Kundu JK, Chun KS (2014). Carnosol induces apoptosis through generation of ROS and inactivation of STAT3 signaling in human colon cancer HCT116 cells. Int J Oncol.

[29] Huang MT, Ho CT, Wang ZY, Ferraro T, Lou YR, Stauber K, Ma W, Georgiadis C, Laskin JD, Conney AH (1994). Inhibition of skin tumorigenesis by rosemary and its constituents carnosol and ursolic acid. Cancer Res.

[30] Johnson JJ, Syed DN, Suh Y, Heren CR, Saleem M, Siddiqui IA, Mukhtar H (2010). Disruption of androgen and estrogen receptor activity in prostate cancer by a novel dietary diterpene carnosol: Implications for chemoprevention. Cancer Prev Res (Phila).

[31] Moran AE, Carothers AM, Weyant MJ, Redston M, Bertagnolli MM (2005). Carnosol inhibits beta-catenin tyrosine phosphorylation and prevents adenoma formation in the C57BL/6J/Min/+ (Min/+) mouse. Cancer Res.

[32] Sotelo-Felix JI, Martinez-Fong D, Muriel De la Torre P (2002). Protective effect of carnosol on CCl(4)-induced acute liver damage in rats. Eur J Gastroenterol Hepatol.

[33] Berman HM, Westbrook J, Feng Z, Gilliland G, Bhat TN, Weissig H, Shindyalov IN, Bourne PE (2000). The Protein Data Bank. Nucleic Acids Res.

[34] Pettersen EF, Goddard TD, Huang CC, Couch GS, Greenblatt DM, Meng EC, Ferrin TE (2004). UCSF Chimera--a visualization system for exploratory research and analysis. J Comput Chem.

[35] Deng N, Goh LK, Wang H, Das K, Tao J, Tan IB, Zhang S, Lee M, Wu J, Lim KH, Lei Z, Goh G, Lim QY (2012). A comprehensive survey of genomic alterations in gastric cancer reveals systematic patterns of molecular exclusivity and co-occurrence among distinct therapeutic targets. Gut.

[36] Daksis JI, Lu RY, Facchini LM, Marhin WW, Penn LJ (1994). Myc induces cyclin D1 expression in the absence of de novo protein synthesis and links mitogen-stimulated signal transduction to the cell cycle. Oncogene.

[37] Lavoie JN, L’Allemain G, Brunet A, Muller R, Pouyssegur J (1996). Cyclin D1 expression is regulated positively by the p42/p44MAPK and negatively by the p38/HOGMAPK pathway. J Biol Chem.

[38] Palmer A, Gavin AC, Nebreda AR (1998). A link between MAP kinase and p34(cdc2)/cyclin B during oocyte maturation: p90(rsk) phosphorylates and inactivates the p34(cdc2) inhibitory kinase Myt1. EMBO J.

[39] Liu F, Rothblum-Oviatt C, Ryan CE, Piwnica-Worms H (1999). Overproduction of human Myt1 kinase induces a G2 cell cycle delay by interfering with the intracellular trafficking of Cdc2-cyclin B1 complexes. Mol Cell Biol.

[40] Parker LL, Piwnica-Worms H (1992). Inactivation of the p34cdc2-cyclin B complex by the human WEE1 tyrosine kinase. Science.

[41] Booher RN, Holman PS, Fattaey A (1997). Human Myt1 is a cell cycle-regulated kinase that inhibits Cdc2 but not Cdk2 activity. J Biol Chem.

[42] Wu CF, Liu S, Lee YC, Wang R, Sun S, Yin F, Bornmann WG, Yu-Lee LY, Gallick GE, Zhang W, Lin SH, Kuang J (2014). RSK promotes G2/M transition through activating phosphorylation of Cdc25A and Cdc25B. Oncogene.

[43] Doehn U, Hauge C, Frank SR, Jensen CJ, Duda K, Nielsen JV, Cohen MS, Johansen JV, Winther BR, Lund LR, Winther O, Taunton J, Hansen SH (2009). RSK is a principal effector of the RAS-ERK pathway for eliciting a coordinate promotile/invasive gene program and phenotype in epithelial cells. Mol Cell.

[44] Xing J, Ginty DD, Greenberg ME (1996). Coupling of the RAS-MAPK pathway to gene activation by RSK2, a growth factor-regulated CREB kinase. Science.

[45] Hutchinson L, Kirk R (2011). High drug attrition rates—where are we going wrong?. Nat Rev Clin Oncol.

[46] Cassidy JW, Caldas C, Bruna A (2015). Maintaining Tumor Heterogeneity in Patient-Derived Tumor Xenografts. Cancer Res.

