# Supplementary material for: Carnosol suppresses patient-derived gastric tumor growth by targeting RSK2
Source: Oncotarget. 2018 Feb 6;9(76):34200–12. doi: 10.18632/oncotarget.24409 (PMC6188138; doi:10.18632/oncotarget.24409)
Supplement: Supplementary file 1 [file oncotarget-09-34200-s001.pdf]

# Carnosol suppresses patient-derived gastric tumor growth by targeting RSK2

## SUPPLEMENTARY MATERIALS

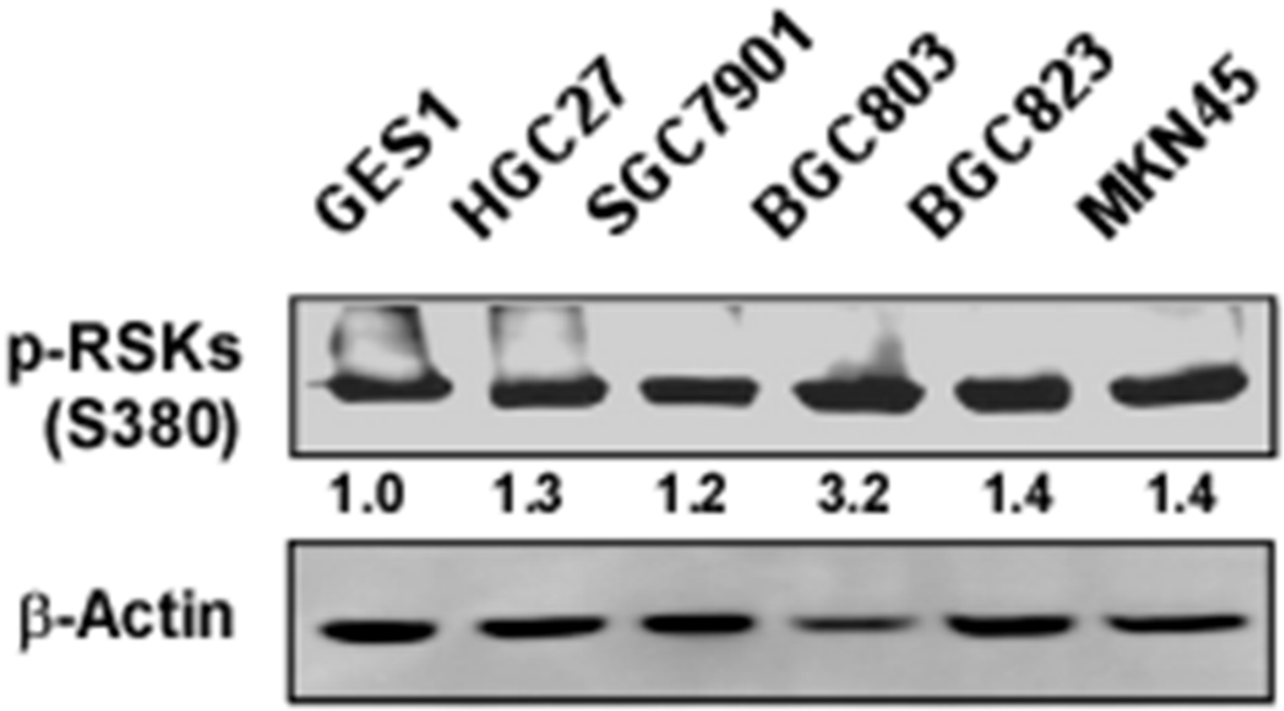


**Supplementary Figure 1: RSK activity in normal gastric cells and gastric cancer cells.** Expression of phosphorylated RSK proteins in normal gastric cells and gastric cancer cells was analyzed by Western blot. Band density was measured using the Image J (NIH) software program.

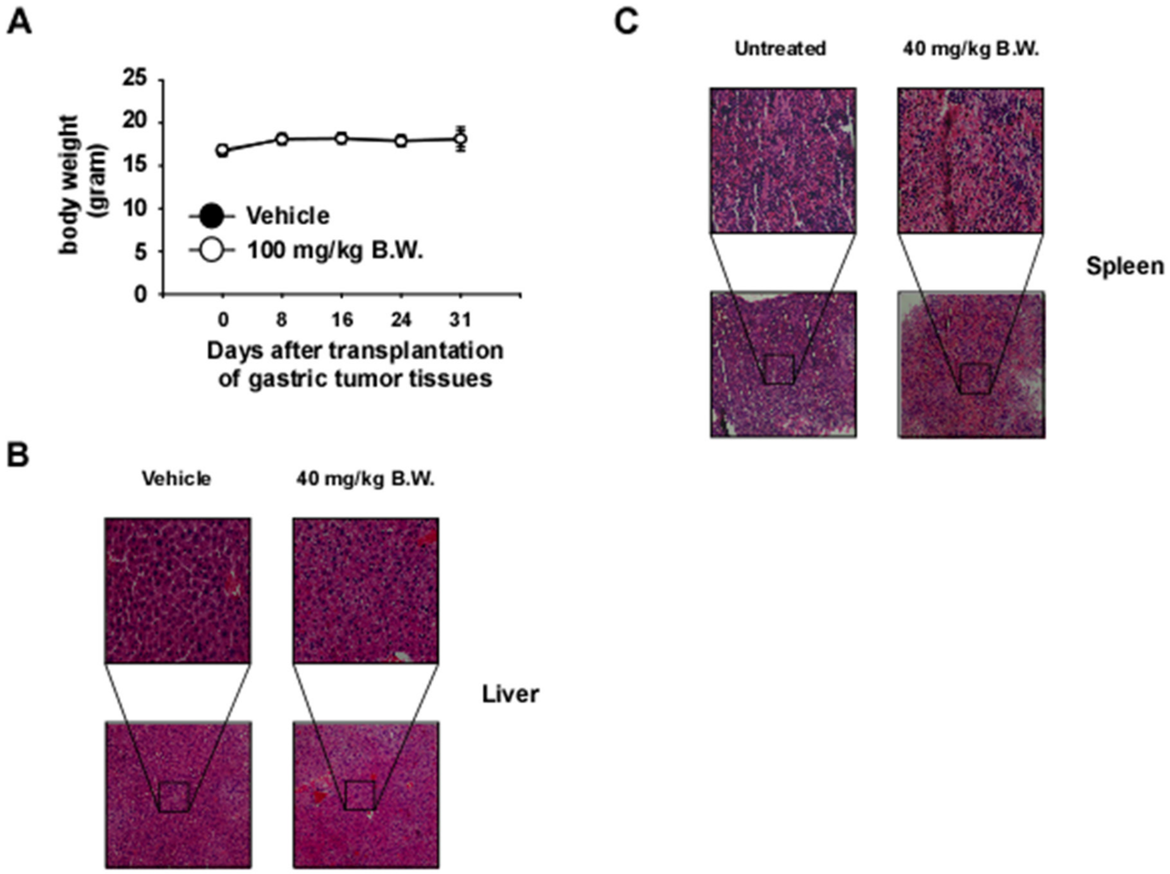


**Supplementary Figure 2: Carnosol has no toxic effects *in vivo*. (A)** Carnosol has no effect on mouse body weight. Body weights from treated or untreated groups of mice were obtained once a week over 31 days. **(B-C)** Immunohistochemistry analysis of liver and spleen tissues. Liver or spleen tissues from treated or untreated groups of mice were stained with H&E.
